# A Comprehensive Review on Steviol Glycosides: Sources, Properties, Bioactivities, Sensory-Functional Enhancement and Bioproduction Strategies

**DOI:** 10.3390/plants15020324

**Published:** 2026-01-21

**Authors:** Liangzhen Jiang, Xun Zhao, Wei Li, Guiru Tang, Yiming Yuan, Jie Cheng, Jun Hua, Liang Zou

**Affiliations:** 1 College of Food and Biological Engineering, Chengdu University, Chengdu 610106, China; 212023086001027@cdu.edu.cn (X.Z.); 212024086001019@cdu.edu.cn (G.T.); chengjie@cdu.edu.cn (J.C.); 2 Sichuan Engineering Technology Research Center of Coarse Cereal Industrialization, Key Laboratory of Coarse Cereal Processing, Ministry of Agriculture and Rural Affairs, Chengdu 610106, China; zouliang@cdu.edu.cn; 3 Sichuan Ingia Biosynthetic Co., Ltd., Chengdu 610200, China; liwei@scingia.com (W.L.); huajun@scingia.com (J.H.); 4 Provincial Key Laboratory of Biotechnology, Key Laboratory of Resource Biology and Biotechnology in Western China, Ministry of Education, College of Life Sciences, Northwest University, Xi’an 710069, China; 202332670@stumail.nwu.edu.cn

**Keywords:** steviol glycoside, biosynthesis, bioactivity, glycosyltransferase, engineering, bioproduction

## Abstract

Steviol glycosides (SGs) are high-intensity, zero-calorie natural sweeteners with demonstrated safety and potential health benefits, positioning them as ideal sucrose substitutes for metabolic disorder management. However, their broad application is limited by inherent drawbacks such as bitterness, low solubility, and inefficient production systems. This review provides a comprehensive summary of recent advances in SG research, covering their sources, properties, and bioactivities. A particular focus is placed on innovative bioproduction strategies—including enzyme engineering, metabolic pathway optimization, and sustainable extraction techniques. Strategies to overcome these challenges through sensory-function enhancement—including formulation and structural modification—are discussed. Furthermore, it highlights emerging trends like microbial chassis-based production and next-generation sweetener design, providing actionable insights for overcoming industrial bottlenecks. By integrating multidisciplinary advances in bioengineering, sensory science, and sustainable processing, this review offers a forward-looking perspective on the development and application of SGs as functional sweeteners in the global food industry.

## 1. Introduction

Sweetness usually brings pleasant feelings to humans [1], which is mainly obtained from dietary sugars. The total global sugar consumption in 2022 is approximately 170 million tons [2]. However, excessive intake of high-calorie sugars, such as sucrose, fructose, and glucose, will lead to metabolic disorders, such as obesity and diabetes, leading to a global health concern [3,4,5]. With rising living standards and increasing health awareness, high-calorie sugars are becoming increasingly substituted by non-nutritive low-calorie sweeteners such as aspartame, saccharin, and acesulfame in food and other fields. However, concerns about the potential side effects of these alternatives have grown over time. Research indicates that extensive use of artificial sweeteners may induce potential health risks, such as increased appetite [6] and potential carcinogenicity [7]. Therefore, natural sweeteners are highly regarded in the market and are ushering in a golden age of rapid growth.

SGs, natural and healthy sweetening alternatives derived from *Stevia rebaudiana*, have garnered increasing consumer interest due to their distinct advantages [8]. As a novel class of terpenoid glycoside compounds, they are 40–400 times sweeter than sucrose [9], containing only 1/300 of its caloric value [10], and exhibiting no toxic side effects. SGs offer distinct advantages over artificial sweeteners, owing to their diverse health-promoting properties such as blood glucose regulation [11], anti-obesity effects [12], antioxidant activity [13], and potential anti-cancer benefits [14]. These attributes align closely with the growing consumer emphasis on health-conscious dietary choices. Nevertheless, the natural content of SGs in stevia plants remains low, necessitating strategies like elicitor treatment and polyploidy induction to enhance biosynthesis [15,16]. Conventional plant extraction methods are insufficient for producing high-purity SGs on a commercial scale, driving the development of advanced biomanufacturing strategies—particularly enzymatic catalysis and microbial synthesis—as critical solutions [17,18]. To provide a systematic review of recent progress, a literature search was conducted across core databases including Web of Science, Scopus, PubMed, Google Scholar, and CNKI, using keywords such as “steviol glycosides,” “enzymatic modification,” “bioactivity,” “microbial synthesis,” and “sensory properties.” Over 200 key publications were selected based on relevance and methodological rigor. This review comprehensively outlines the current research status of SGs, covering their botanical sources, structural and physicochemical properties, biological effects, and in vivo biosynthetic pathways. It highlights recent advances in SG production, including enzymatic synthesis and whole-cell catalysis using engineered microorganisms, along with optimized metabolic engineering strategies. Ongoing research continues to reveal promising prospects for improving production efficiency and expanding applications in the food and pharmaceutical industries.

## 2. The Natural Source of SGs

**Figure 1:**
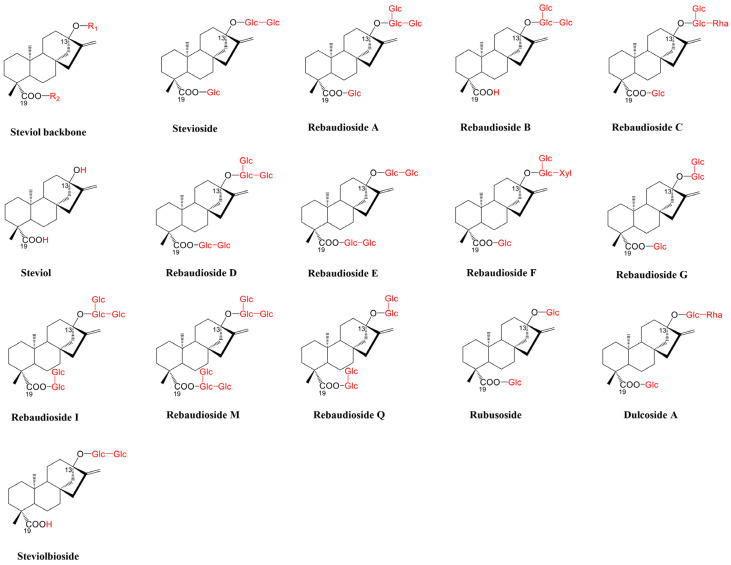
The structures of steviol moiety and SGs. The highlighted red groups are the sites of glycosylation. Glc, glucose; Xyl, xylose; Rha, rhamnose.

*Stevia rebaudiana* is the viable source plant of SGs. There are over 200 species of Stevia worldwide, but *S. rebaudiana*, due to its rich content of sweet compounds, namely, SGs, is the only one with a sweet taste [13]. *S. rebaudiana* possesses a shallow, extensive root system and branched, erect stems, typically reaching 60–70 cm in cultivation [19]. Its sessile, opposite leaves are elliptical to lanceolate with serrated margins, and the shoots are slightly pubescent. The plant produces characteristic small white *Asteraceous* flowers arranged in capitula and panicles, yielding slender, pappus-bearing achenes [19]. SGs are glycosylation derivatives of steviol (Figure 1) [20], a diterpene mainly found in leaves of *S. rebaudiana*. The two most abundant SGs, stevioside (Stv), and rebaudioside (Reb) A, totally account for 7–15% of the total weight of dry leaves, depending on the genotype, the cultivation, and harvesting conditions of *S. rebaudiana* [3]. In addition, *S. rebaudiana* also contains trace amounts of Reb B-F, and M, I, and Steviolbioside (SB). *S. rebaudiana*, indigenous to South America, is cultivated in many regions globally nowadays [21]. Internationally, Brazil ranks as one of the largest producers, in terms of both cultivation area and output [22]. China is a significant producer and the largest exporter of *S. rebaudiana* in the world. The plant is primarily grown in Yunnan, Guizhou, Guangxi, and other regions of China, where unique climatic and soil conditions contribute to high yield. Paraguay also plays a crucial role in producing recognized high-quality *S. rebaudiana* [23]. Countries like Japan, South Korea, and Vietnam also have substantial *S. rebaudiana* cultivation, mainly to meet domestic demands and to some extent for export purposes [22].

## 3. Structure and Properties of SGs

To date, over 60 SGs have been identified in *S. rebaudiana* [24]. The structures of some of the most focused on SGs are displayed in Figure 1. The SGs share the common tetracyclic diterpenoid aglycon, steviol. By connecting different numbers of glucose, rhamnose, or xylose units through an ester bond at positions C-13 or C-19 with β-1,2 or -1,3 glycosidic bonds, various SGs with different tastes are formed, including SB, Rubusoside (Rub), dulcoside A, Stv, Reb A-F, Reb I, and Reb M (Figure 1). Among them, Rub and SB are diglycosyl. Stv, Dulcoside A, Reb B, and Reb G are triglycosyl. Reb A, C, E, F, and Q are tetroglycosyl, while Reb D and I are pentaglycosylated. Reb M bears the most sophisticated hexaglycosylation structures, with a branched tri-glycosyl unit both at C-13 and C-19. The specific glycosylation structures at C-13 and C-19 of SGs are illustrated in Table 1.

The shared properties of SGs are pure white/yellow color, with tastes close to sucrose, and no odor. The properties of SGs are illustrated in Table 2. Rub is 114 times sweeter than sucrose, with a slight aftertaste [25]. Due to the rhamnose group in Dulcoside A, it has a significant bitter aftertaste, and its value is much lower than Stv and Rub [26]. Stv and Reb A exhibit approximately 300 times and 450 times the sweetness of sucrose, respectively. Reb A was first isolated from *S. rebaudiana* leaves in the 1970s [27]. It can be enzymatically transformed into various other glycosides, making it a key substrate in the microbial synthesis of SGs [28]. Thus, Reb A is also considered to be the most commercially significant compound in the biosynthesizing pathway of SGs [28]. As predominant SGs in *S. rebaudiana*, Stv and Reb A have been commercialized with extensive applications as sugar substitutes in beverages and food products [29]. Nevertheless, neither the tastes of Stv nor Reb A are satisfactory for the food industry, with a characteristic bitter aftertaste, or a ‘‘licorice’’ taste. Reb B is approximately 150 times the sweetness of sucrose, yet its solubility at room temperature is only 0.01–0.02% [28]. Reb D is 200–350 times sweeter than sucrose and shows a quicker sweetness onset with reduced aftertaste compared to Reb A and Stv [30,31], thus with higher potential commercial values. However, its poor water solubility and low content in *S. rebaudiana* (0.42–0.5% of dry weight) pose challenges for extraction [32]. Reb E, with approximately 150–300 times the sweetness of sucrose [9], serves as a precursor for the biosynthesis of Reb D and Reb M. Reb M, 200–350 times sweeter than sucrose, provides rapid sweetness and sensory characteristics most comparable to sucrose, with less bitterness and astringency compared to Reb A [30,33]. It is sparingly soluble in water, exhibiting stability similar to Reb A [34]. Reb M has been recognized as the next generation SG due to its overall taste quality, which mimics the fast, clean sweetness of sucrose [28]. Reb I is a natural non-cyclic sweetener. Ohta et al. first isolated Reb I from stevia, deriving it through conversion from Reb A [35]. Novel SGs bearing alternative glycosyl groups, such as rebaudioside FX1 (Reb FX1), display optimized sweetener characteristics, indicating promising commercial adaptability in processed foods [36].

## 4. The Biosafety and Functional Effects of SGs

### 4.1. Toxicity and Safety

Extensive toxicological evaluations support the general safety of SGs and their enzymatically or microbially produced derivatives for use as food sweeteners [41,46,51,52,53]. The metabolic safety of major glycosides like STV, Reb A, Reb D, and Reb M is well-established through in vitro tests by fecal homogenate from adults and children, as they are efficiently hydrolyzed to steviol by gut microbiota and excreted [54,55,56,57]. No evidence indicates genotoxicity [53,58,59] or carcinogenicity [53,58], with studies showing no DNA damage or adverse effects in long-term rodent bioassays at high doses (up to 2000 mg/kg steviol equivalents) of Reb D [41] or Reb M [46,56,60,61,62]. No metabolic side effects were observed after oral administration of high doses of SGs in patients or healthy people in limited clinical trials [63,64]. Daily consumption for 4 weeks of a beverage sweetened with stevia (at 25% of the ADI), compared to one sweetened with 30 g of sucrose, showed no significant effects on the gut microbiome, fecal short-chain fatty acids (SCFA), or fasting cardiometabolic parameters in adults [64]. The Food and Drug Administration (FDA) granted stevia extract the GRAS status in 2018 [65]. International regulatory bodies, including the Joint FAO/WHO Expert Committee on Food Additives (JECFA) and the European Food Safety Authority (EFSA), have affirmed the safety of STV, Reb A, Reb D [66], and Reb M [61,62,67,68,69,70] for use as general-purpose sweeteners without concerns. The National Health Commission of the People’s Republic of China (NHC) approved stevia-extracted SGs (>95% purity) as food additives in 2020 [71]. EFSA has set a unified Acceptable Daily Intake (ADI) of 0–4 mg/kg body weight per day (expressed as steviol equivalents) for SGs with purity higher than 95% [59,70].

Evidence regarding the reproductive safety of SGs is nuanced. While traditional use and regulatory assessments have not indicated teratogenicity [59,72,73], studies on specific glycosides like Reb A report conflicting outcomes, showing no adverse effects in some studies [74] but potential alterations in aged animal models in others [75]. These discrepancies likely reflect variables such as glycoside purity, dosage, and physiological context, underscoring the need for further standardized investigation.

### 4.2. Anti-Diabetic Effects

**Figure 2:**
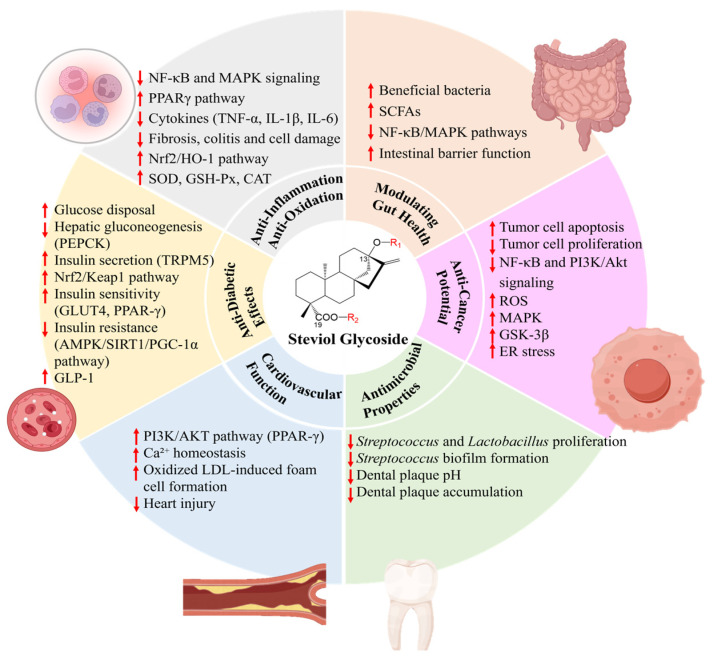
Schematic representation of the potential functional effects of SGs. Red arrows represent activation/positive regulation (upwards) or inhibition/negative regulation (downwards) of the pathways or the compounds.

SG, primarily Stv and Reb A, exhibit multi-target anti-diabetic properties through complementary mechanisms (Figure 2) that enhance glucose homeostasis, as evidenced in preclinical studies. The most well-defined action is the potentiation of glucose-stimulated insulin secretion from pancreatic β-cells via TRPM5 activation in cells [76,77] or in mice [77], independently of K(ATP) channel activity or intracellular cAMP levels [76,77]. Beyond insulin secretion, SGs contribute to glycemic control by inhibiting hepatic gluconeogenesis through downregulation of phosphoenolpyruvate carboxykinase (PEPCK) [78] and enhancing glucose disposal in peripheral tissues like skeletal muscle in rats [79]. Additionally, SGs activate the AMPK/SIRT1/PGC-1α pathway to improve mitochondrial function, reduce oxidative stress, and ameliorate insulin resistance and muscle fiber degeneration in diabetic mouse models [80]. They also regulate genes involved in glucose and lipid metabolism, and improve blood lipid profiles [81], provide renal protection via Nrf2/Keap1 pathway modulation to reduce oxidative stress in diabetic rat kidneys [82], and target GLUT4 and PPAR-γ to enhance insulin sensitivity [82,83] in obesity-related metabolic disorders in rats. Reb A further stimulates GLP-1 release from intestinal enteroendocrine cells, potentially through bitter taste receptor (TAS2R) signaling, augmenting insulin secretion [84]. Reb M was associated with improved insulin sensitivity and reduced weight gain under obesogenic conditions in a 20-week mouse study [46].

Clinical evidence, though limited by a paucity of high-quality randomized controlled trials (RCTs), generally supports a beneficial role of SGs in glycemic regulation [85], consistent with their historical use in diabetes management among Indians. In individuals with type 2 diabetes (T2D), an acute dose of Stv (1 g) reduces postprandial blood glucose and improves the insulinogenic index [86,87]. A meta-analysis indicates SG consumption can lower fasting blood glucose (FBG) in certain adult subgroups, despite limited evidence quality and heterogeneity among studies being noted [88]. Acute intake of stevia lowers postprandial glucose and insulin without triggering compensatory overeating [89]. However, findings are not uniformly consistent. A separate meta-analysis of RCTs reported a significant reduction in systolic blood pressure with SG consumption, but did not find significant effects on FBG, glycated hemoglobin (HbA1c), body mass index, diastolic blood pressure, or lipid profiles, with notable heterogeneity across studies [87]. Importantly, the observed glucoregulatory effects appear to be context-dependent. They are evident in the context of T2D but are absent in T1D, where SG consumption is nonetheless not associated with adverse effects [63].

### 4.3. Anti-Inflammation and Anti-Oxidation

Common SGs possess notable anti-inflammatory and antioxidant properties by modulating conserved signaling pathways preclinically. The anti-inflammatory effect is primarily attributed to the suppression of pro-inflammatory cascades. In vitro studies show that Stv and Reb A inhibit the activation of the NF-κB and MAPK pathways in immune cells (e.g., macrophages) [90,91], chondrocytes [37], intestinal epithelial cells [92], and human colon carcinoma cells [93], reducing the expression of cytokines like TNF-α and IL-6 [90,92,93], and enzymes such as iNOS and COX-2 (Figure 2) [37]. This action is complemented by the activation of cytoprotective pathways, including the upregulation of the Nrf2/HO-1 antioxidant axis [37] and the enhancement of PPARγ activity [94]. In vivo, these mechanisms translate to protective effects in various animal models of disease. Stv administration ameliorates intestinal inflammation in models of colitis [95,96,97], protects against LPS-induced systemic inflammation and organ injury [98], alleviates osteoarthritis [99,100] by inhibiting chondrocyte inflammation and cartilage degradation [99], and shows efficacy in adjuvant-induced arthritis [101]. Other glycosides exhibit targeted activities: Rub mitigates neuroinflammation in SN4741 cells [102], Reb B modulates cytokines in lung injury cell and rat models [39], and Reb D/Reb M8 inhibit TNF-α in cells [43]. The antioxidant properties, closely linked to anti-inflammatory actions, are largely mediated through Nrf2 pathway activation. Treatment with SGs enhances the activity of endogenous antioxidant enzymes (e.g., SOD, GSH-Px, CAT) and reduces markers of oxidative damage (e.g., MDA, ROS) in both cellular and animal models [92,94,103,104].

### 4.4. Modulating Gut Health

SGs could beneficially modulate gut health preclinically (Figure 2). Rub treatment modulates the gut microbiome and metabolome in Parkinson’s disease mice [102]. Reb D enriched beneficial bacteria (*Faecalibacterium rodentium*), improved bile acid metabolism, and reduced markers of metabolic endotoxemia [41], whereas Reb M specifically increased the prevalence of colonic *Lachnospiraceae* bacteria in mouse models [46]. In vitro, Stv fermentation increased SCFA production [105], and in intestinal epithelial cells (IPEC-J2), it protected intestinal barrier function and reduced inflammation via NF-κB/MAPK pathways [92]. In contrast, a 4-week human RCT found that SG consumption at a moderate dose did not significantly alter gut microbiome composition or SCFA levels in healthy adults, though it prevented a slight BMI increase seen with sucrose [64].

### 4.5. Cardiovascular Function

SGs exhibit potential cardiovascular benefits through diverse mechanisms (Figure 2). In vitro, Ru protects neuronal cells by inhibiting apoptosis and inflammation via modulation of JNK, p38 MAPK, and NF-κB pathways [102]. In rodent models, Stv provides neuroprotection against cerebral ischemia by activating the PI3K/AKT pathway [106,107] and ameliorates heart injury by reducing oxidative stress and modulating calcium homeostasis [94,108], potentially through the NF-κB/TGF-β1/Smad pathway [109]. SGs and related diterpenoids also inhibit oxidized LDL-induced foam cell formation, suggesting anti-atherosclerotic activity [110,111]. The blood pressure-lowering effects of SGs are inconsistent, showing dependency on administration routes and specific compounds. In animal models, intravenous administration of Stv lowered blood pressure dose-dependently [112,113], while oral Reb A showed no effect [114]. Human clinical data are also variable, with one long-term study reporting significant reductions [115,116] and another shorter trial finding no significant difference from placebo [117].

### 4.6. Anti-Cancer Potential

SGs have garnered significant attention in cancer therapy due to their high efficacy and safety. Stv inhibits hepatocellular carcinoma by modulating NF-κB and PI3K/Akt signaling (Figure 2) [118], and induces apoptosis in gastrointestinal, breast, and bladder cancer cells through mechanisms involving ROS generation, MAPK signaling, and ER stress pathways [119,120,121,122,123,124]. Reb A and some steviol or isosteviol derivatives also trigger caspase-dependent apoptosis in different cancer cells [125,126]. Notably, their anti-cancer efficacy can be enhanced through advanced delivery systems [125]. In vivo studies support these findings, showing Stv can inhibit liver tumor growth and reduce breast adenoma incidence in rats without promoting carcinogenesis in long-term studies [57,118].

### 4.7. Antimicrobial Properties

SGs display notable antimicrobial properties (Figure 2). In vitro, Stv shows bacteriostatic effects against microorganisms like *Escherichia coli*, *Staphylococcus aureus*, and *Candida albicans* [127,128,129]. A prominent application is in oral health [129,130,131,132]. In vitro, stevia leaf extracts inhibit susceptible microorganisms [129], and 10% solutions of Stv or Reb A reduce *Streptococcus mutans* biofilm formation [130]. In vivo human studies confirm that Stevia extract rinses elevate plaque pH and reduce plaque accumulation compared to sucrose [131,132]. Research into enhancing this activity includes using Stevia extracts to synthesize nanoparticles with broad-spectrum effects, potentially offering alternatives against multidrug-resistant pathogens [133,134].

### 4.8. Summary on Limitations and Gaps in Clinical Validation of SG Activities

Despite promising preclinical findings, the clinical evidence for specific therapeutic benefits of SGs remains limited, constrained by key gaps. A primary limitation is the lack of large-scale, long-term RCTs, as most human studies involve small, heterogeneous cohorts over short durations, limiting the robustness and generalizability of results. Reported outcomes are sometimes inconsistent; for instance, hypotensive [115,116,117] and glycemic effects in type 2 diabetes [87] vary considerably with the specific compound and administration route, and no glycemic benefit is observed in type 1 diabetes, highlighting the context-dependent mechanisms tied to β-cell function [63,64]. Reported outcomes are often inconsistent; for example, clinical evidence is virtually absent for many other purported bioactivities (e.g., direct neuroprotection, most anti-cancer effects). Furthermore, studies frequently rely on surrogate biomarkers rather than definitive clinical endpoints, and the long-term physiological relevance of short-term intervention effects (e.g., on gut microbiota) remains unknown [63]. Consequently, robust, targeted RCTs with clinically relevant outcomes in appropriate populations are urgently needed to validate the potential therapeutic applications of SGs.

## 5. Natural Biosynthesis of SGs

### 5.1. Natural Biosynthetic Pathway of SGs

**Figure 3:**
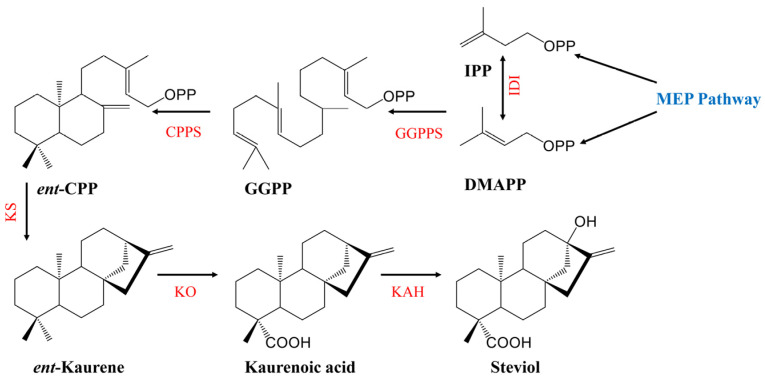
The biosynthetic pathway of steviol. This figure shows the two-stage cellular process to produce steviol, the core backbone of all SGs. The first stage generates the basic building blocks (IPP and DMAPP) for terpenoids from the methyl erythritol phosphate (MEP) pathway. The second stage assembles these blocks through a series of enzymatic steps, ultimately forming the key intermediate, steviol. IDI: isopentenyl pyrophosphate isomerase; GGPPS: geranyl geranyl diphosphate synthase; CPPS: copalyl diphosphate synthase; KS: kaurene synthase; KO: kaurene oxidase; KAH: kaurenoic acid hydroxylase; IPP: isopentenyl pyrophosphate; DMAPP: dimethylallyl pyrophosphate; GGPP: geranylgeranyl diphosphate; CPP: copalyl diphosphate. OPP: pyrophosphate group. The red color indicates the enzymes that catalyze the reactions, while the blue color represents the entire MEP pathway.

**Figure 4:**
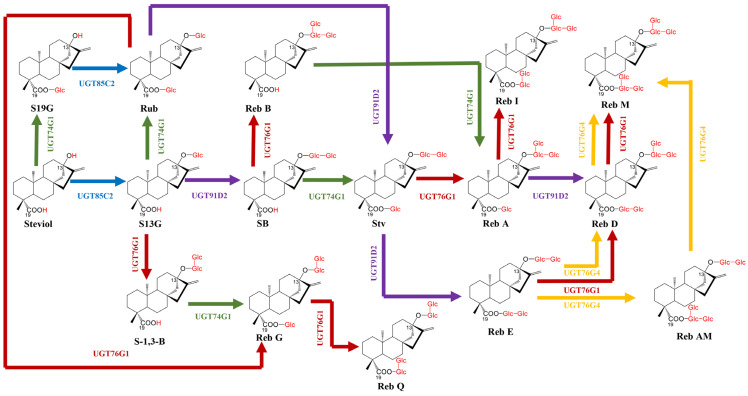
The glycosylation steps involved in SG biosynthesis. This diagram outlines the biosynthetic pathway for key SGs. It shows how the core structure, steviol, is progressively modified by specific glycosyltransferases that attach sugar units, leading to the production of different SGs, like Stv, Reb A, and Reb M. Stv: stevioside; SB: steviolbioside; Rub: rubusoside; S13G: steviolmonoside; S-1,3-B: steviol-1,3-bioside; S19G, Steviol-19-O-monoglucoside; Reb A, Rebaudioside A; Reb B, Rebaudioside B; Reb D, Rebaudioside D; Reb E, Rebaudioside E; Reb G, Rebaudioside G; Reb I, Rebaudioside I; Reb M, Rebaudioside M; Reb AM, Rebaudioside AM. Arrows of different colors represent distinct enzymatic reactions, and the words in corresponding colors denote the enzymes participating in each respective reaction.

The biosynthetic pathway of the most focused on SGs in *S. rebaudiana* has been elucidated [135], and can be divided into three distinct modules. The first module, localized within plastids, involves the methyl erythritol phosphate (MEP) pathway generating the universal terpenoid precursors isopentenyl diphosphate (IPP) and dimethylallyl diphosphate (DMAPP) [136,137]. In the second module, occurring in the endoplasmic reticulum, IPP and DMAPP are sequentially converted to *ent*-kaurenoic acid (*ent*-KA). This process initiates with the condensation of IPP and DMAPP into geranylgeranyl diphosphate (GGPP) catalyzed by GGPP synthase (GGPPS). GGPP is then converted to *ent*-copalyl diphosphate (*ent*-CPP) by CPP synthase (CPPS), followed by cyclization and carboxylation steps mediated by the cytochrome P450 enzymes kaurene synthase (KS) and kaurene oxidase (KO) to yield *ent*-KA [138] (Figure 3). The third module, taking place in the cytoplasm, features the pivotal hydroxylation of *ent*-KA at the C-13 position by kaurenoic acid hydroxylase (KAH, a CYP450 enzyme), forming steviol as the core aglycone for SGs; this step represents a branch point diverging from gibberellin (GA) biosynthesis. Subsequently, various UDP-dependent glycosyltransferases (UGTs) catalyze the attachment of differing numbers and types of sugar moieties to the C-13 hydroxyl and C19 carboxyl groups of steviol, resulting in the diverse array of SGs (Figure 4) [20].

### 5.2. Glycosyltransferases Involved in the Biosynthesis of SGs

In *S*. *rebaudiana*, the structural diversification of SGs is exclusively mediated by UGTs [139], predominantly classified under Glycosyltransferase Family 1 (GT1) in the CAZy database [140]. These Leloir-type enzymes utilize UDP-sugar donors to catalyze stereospecific glycosylation, with a conserved C-terminal PSPG (Plant Secondary Product Glycosyltransferase) motif serving as the critical binding domain for UDP-glucose recognition [141]. The biosynthesis is orchestrated in vivo by four core UGTs with well-defined catalytic roles (Table 3) [142]. UGT85C2 initiates β-1-glucosylation at the C-13-OH of steviol to form steviolmonoside (S13G). UGT74G1 specifically modifies the C19-COOH, converting substrates like steviol to Steviol-19-O-β-D-glucoside (S19G) and S13G to Rub. UGT91D2 catalyzes β-1,2-glycosylation at C-13 or C-19 positions, producing compounds such as SB from S13G and Stv from Rub, and cannot catalyze β-1,2-glycosylation when a β-1,3-linked disaccharide already occupies the targeted positions. UGT76G1 performs β-1,3-glucosylation at either position, with broad substrate specificity, including Rub, S13G, SB, Reb G, Stv, Reb A, Reb D, and Reb E [143], enabling key steps like the conversion of Stv to Reb A, and Reb D to Reb M [139,144,145]. The characteristic substrate promiscuity of UGTs [146,147] allows for the recognition of multiple sites on SG intermediates, enabling a complex network that generates diverse final products with varying sweetness profiles and physicochemical properties. The biosynthesis of major SGs involves defined routes (Figure 4): steviol is first glycosylated by UGT85C2 or UGT74G1 to form S13G or S19G, which converge to Rub; UGT91D2 then converts S13G to SB or Rub to Stv, and UGT74G1 transforms SB to Stv; UGT76G1 glycosylates Stv to Reb A, and downstream [148,149], Reb D is generated from Reb A via UGT91D2 or directly from Stv via Reb E, with UGT76G1 ultimately converting Reb D to Reb M [142,148,149]. Notably, UGT76G1 also drives side reactions from SB to Reb A and from Rub to other glycosides like Reb G/Q, making it and UGT91D2 the primary engines shaping the SG profile [150]. A critical functional variant, UGT76G4, exhibits strong C19-position preference for β-1,3-glucosylation, making it efficient for Reb M synthesis from Reb D [143,151]. This advantage establishes UGT76G4 as a promising key enzyme for the targeted biosynthesis of rare, high-value sweeteners like Reb M.

Beyond endogenous UGTs, heterologous enzymes from other species offer alternatives for SG synthesis (Table 3). UGTs such as *At*UGT73C1 (from *Arabidopsis thaliana*) [152], *Rs*UGT85A57 (from *Rubus suavissimus*) [153] and *Ak*UGT85A58 (from *Angelica keiskei*) [154] mimic UGT85C2’s C-13-OH activity, while *Sr*UGT73E1 [155], *Rs*UGT75L20 and *Rs*UGT75T4 [153,154], and *Ak*UGT75L21 and *Ak*UGT75W2 [154] mirror UGT74G1’s C-19-COOH activity. *Os*UGT91C1 (from *Oryza sativa*) functionally analogizes UGT91D2 [156], and enzymes like *Sl*UGTSL2 (from *Solanum lycopersicum*) [157], *Pg*UGT (from *Panax ginseng*) [158], and *St*UGT (from *Solanum tuberosum*) [159] catalyze Reb A to Reb D conversion. Notably, the bacterial glycosyltransferase *Bs*YojK (from *Bacillus subtilis* 168) efficiently performs β-1,2-glucosylation of Reb A to Reb D, showing industrial promise [160]. However, a significant gap remains in identifying heterologous enzymes with UGT76G1-like activity for efficient Reb M production, highlighting a key area for future enzyme engineering. Collectively, the substrate promiscuity of these UGTs enables a complex network that generates diverse SGs with varying properties, but their regioselectivity and expression limitations necessitate further research to optimize heterologous biosynthesis.

**Table 3:** The UGTs involved in the enzymatic synthesis of SGs and glucosylated steviol glycosides (GSGs).

Type	Enzymatic Reaction	Protein	Source Organism	Phylum	Substrate	**Product**	**Reference**
SG	β-1-glucosylation at the C-13 hydroxyl position	*Sr*UGT85C2	*Stevia rebaudiana*	Plant	Steviol, S19G	S13G, Rub	[3,144]
		*At*UGT73C1	*Arabidopsis thaliana*	Plant	Steviol	S13G	[152]
		*Rs*UGT85A57	*Rubus suavissimus*	Plant	S19G	Rub	[153]
		*Ak*UGT85A58	*Angelica keiskei*	Plant	Steviol, S19G	S13G, Rub	[154]
SG	β-1-glucosylation at the C-19 carboxyl position	*Sr*UGT74G1	*Stevia rebaudiana*	Plant	Steviol, S13G, SB, S-1,3-B, Reb B	S19G, Rub, Stv, Reb G, Reb A	[3,144]
		*Sr*UGT73E1	*Stevia rebaudiana*	Plant	S13G	Rub	[155]
		*Rs*UGT75L20	*Rubus suavissimus*	Plant	Steviol, S13G	S19G, Rub	[153,154]
		*Rs*UGT75T4	*Rubus suavissimus*	Plant	Steviol, S13G	S19G, Rub	[153]
		*Ak*UGT75L21	*Angelica keiskei*	Plant	Steviol, S13G	S19G, Rub	[154]
		*Ak*UGT75W2	*Angelica keiskei*	Plant	Steviol, S13G	S19G, Rub	[154]
SG	β-1,2-glucosylation of the C-13 or C-19 position	*Sr*UGT91D2	*Stevia rebaudiana*	Plant	Rub, S13G, Reb A, Stv	Stv, SB, Reb D, Reb E	[145,161,162]
	β-1,2-glucosylation of the C-19 position	*Pg*UGT	*Panax ginseng*	Plant	Reb A	Reb D	[158]
SG or GSG	β-1,2 or β-1,6-glucosylation at the C-19 position	*Os*UGT91C1	*Oryza sativa*	Plant	Reb A, Reb E	Reb D, Reb D2′	[156,163,164]
SG or GSG	β-1,2 or β-1,6-glucosylation at the C-13 position	*Sl*UGTSL2	*Solanum lycopersicum*	Plant	Reb A, Reb D	Reb D, Reb M2	[49,165]
SG	β-1,2-glucosylation at the C-19 position	*Bs*Yojk	*Bacillus subtilus* 168	Bacteria	Reb A	Reb D	[160]
SG		*St*UGT	*Solanum tuberosum*	Plant	Reb A	Reb D	[159]
SG	β-1,3-glucosylation at the C-13 or C-19 position	*Sr*UGT76G1	*Stevia rebaudiana*	Plant	Reb D	Reb M	[3,144]
		*Sr*UGT76G4	*Stevia rebaudiana*	Plant	Reb D	Reb M	[151]
					Reb E	Reb M	
GSG	β-1,6-glucosylation at the C-13 position	*Gj*UGT94E13	*Gardenia jasminoides*	Plant	Reb D, Reb M2	Reb M8, Reb M9	[43,50,166]
		*Bs*YjiC	*Bacillus subtilis* 168	Bacteria	Reb A	Reb L2	[167]
	β-1,6-glucosylation at the C-19 position	*Si*UGT94D1	*Sesamum indicum*	Plant	Reb A, Reb D	Reb D2, Reb M2	[168,169]
		*Nt*UGT	*Nicotiana tabacum*	Plant	Reb D	Reb M2	[170]

## 6. Methods to Obtain SGs

### 6.1. Enhancement of SG Biosynthesis in Planta

#### 6.1.1. Strategies for Enhanced In Vivo Production

The biosynthesis of SG in *S. rebaudiana* is influenced by a range of physiological and genetic factors. Elicitor application represents an effective strategy to stimulate both plant growth and SG accumulation. Environmentally friendly oligosaccharide elicitors—such as chitosan oligosaccharides (COS, 50–100 mg/L) and alginate oligosaccharides (AOS, 50 mg/L)—enhance biomass and SG production by improving photosynthetic efficiency [171]. Similarly, treatment with chitosan (200 mg/L) and methyl jasmonate (100 mg/L) significantly elevates Reb A and Stv content, respectively [172]. Beneficial microbial inoculation and endophytic bacteria further promote SG accumulation and plant resilience, reducing dependency on chemical fertilizers [173]. Plant growth regulators also modulate SG biosynthesis. While the retardant CCC (2-chloroethyltrimethylammonium chloride) suppresses plant height, paclobutrazol (PBZ) at 12 ppm enhances not only growth but also SG production and antioxidant capacity [174]. Polyploidy induction has emerged as a powerful breeding tool for SG enhancement. Treatment of germinating seeds with colchicine (0.05% for 48 h or 0.1% for 24 h) successfully induces tetraploid lines, which exhibit elevated Stv and Reb A levels alongside distinct morphological traits [16,175]. Integrated transcriptomic and metabolomic analyses reveal that polyploidization upregulates key SG biosynthesis genes, correlating with enhanced glycoside accumulation [176]. These findings support polyploid breeding as a promising strategy for developing high-yielding *Stevia* varieties.

#### 6.1.2. Advances in Plant Extraction Methods

Although microbial production of SGs is advancing, plant extraction remains the primary industrial source, mainly yielding Stv and Reb A. Conventional techniques such as steam distillation and boiling often lack specificity and are inefficient in recovering minor but valuable glycosides such as Reb D and Reb M [72]. Recent efforts focus on sustainable and efficient extraction technologies. Supercritical fluid extraction (SFE) using CO_2_ offers high selectivity for thermolabile SGs with minimal environmental impact [177]. Pressurized hot water extraction (PHWE) enables rapid, solvent-free recovery of non-polar glycosides and is readily scalable [178]. Natural deep eutectic solvents (NADES) serve as tunable, bio-based media; for example, choline chloride–urea systems improve Reb A recovery while reducing toxicity [179]. When combined with ultrasound-assisted extraction (UAE), NADES further enhances Stv and Reb A yields by more than threefold compared to conventional methods, leveraging cavitation to disrupt cell walls while cutting solvent use by 40–60% [180]. Nevertheless, the low natural abundance of Reb D and Reb M (<1% of leaf glycosides) continues to challenge direct extraction. Although metabolic engineering and modulation of glycosylation pathways in planta show potential for increasing their biosynthesis, such strategies remain largely confined to laboratory-scale development.

### 6.2. Biosynthesis of SGs In Vitro

Traditional methods for extracting SGs from Stevia are inefficient, resulting in a complex mixture with astringent and bitter flavors that limit its applications [3]. The same applies to chemical synthesis. Since the chemical structures of SGs are complex, their chemical synthesis is intricate, typically involving multiple steps. Chemical synthesis often yields low production due to the formation of intermediates and by-products [181]. Moreover, this method poses safety risks and environmental pollution concerns. Therefore, chemical synthesis is considered a novel approach but has not been widely adopted due to these limitations. With the increasing demand for SGs and their low concentration in Stevia, especially Reb D and Reb M, traditional plant extraction and purification methods could not meet the market effectively, necessitating the development of rational new synthetic approaches for SGs.

#### 6.2.1. Coupling Enzyme Reaction

Enzymatic Catalysis and Enzyme Engineering

Enzymatic synthesis represents a pivotal green strategy for the precise and sustainable production of SGs, leveraging its hallmark high regioselectivity and operation under mild conditions [182,183]. The core of this technology resides in glycosyltransferases (UGTs), whose performance is critically dependent on catalytic efficiency and substrate specificity. Protein engineering techniques—including rational design, directed evolution, and semi-rational design—have been extensively employed to enhance UGT activity, thermostability, and product specificity [182,183].

Engineering for Targeted Glycosylation

The enzymatic synthesis of key SGs primarily utilizes abundant precursors like Stv and Reb A. For instance, the conversion of Stv to Reb A has been achieved via in situ transglycosylation in leaves, enriching content from 4% to 66% [184], or through in vitro catalysis with β-1,3-glucanase, yielding a 62.5% conversion rate [185]. Enzymatic synthesis of Reb D predominantly utilizes the abundant precursor Reb A, with enzyme engineering serving as the key strategy to enhance catalytic efficiency, regioselectivity, and thermal stability for industrial scalability (Table 4). Initial efforts focused on UGT91D2, where the V155T mutation improved UDPG binding and yield [42,186,187]. Broader engineering targeted versatile glycosyltransferases: *Os*UGT91C1 was redesigned (F379A/F208M) to achieve exclusive β-1,2 activity for specific Reb D synthesis [156,188], while *Sl*UGTSL2 optimization (N358F) boosted yield [157]. Semi-rational design of *Pg*UGT yielded a multi-site variant with improved activity and thermostability [158]. Engineering of the bacterial glycosyltransferase *Bs*YojK has generated highly efficient and stable variants. The I241T/G327N double mutant achieved a high conversion rate of over 91% for the synthesis of Reb D (Table 4) [160]. Notably, the introduction of the S158E/A218H/A369K triple mutation dramatically enhanced thermostability, increasing the enzyme’s half-life at 50 °C from 2.16 h to 58.64 h while maintaining a high catalytic yield [189]. Additional stabilizing mutations, such as Q251M and R366P, have also been shown to improve the enzyme’s performance at elevated temperatures [190]. These advancements, driven by improved substrate binding and structural rigidity, underscore the strong potential of engineered biocatalysts for cost-effective Reb D production. To enhance the enzymatic synthesis of Reb M from Reb D, extensive engineering of UGT76G1 has been pursued to overcome its broad substrate specificity and enhance its performance in synthesizing Reb M from Reb D. Key mutations, such as S195Q and T284S, have been identified to improve catalytic efficiency and product specificity [135,148]. Further optimizations, including the double mutant L200A/L379M (Table 4), have achieved substantial activity increases (10-fold) and high product yields (96.85%) [191,192]. Thermostability has also been improved, as demonstrated by multi-point mutants that retain high yields at elevated temperatures [193]. Beyond UGT76G1, engineering of the C19-preferring UGT76G4 has generated hyperactive variants (H155S/Q199I/G200Y) with significantly enhanced activity (23-fold) [151]. Moreover, integrating engineered UGT94B1 into cascade systems has enabled efficient, high-titer (38.8 g/L) production of Reb M from simpler substrates, showcasing its potential for industrial-scale synthesis [194].

Enzyme Immobilization for Process Sustainability

To address challenges like enzyme cost and reusability, immobilization offers an effective strategy. For example, co-immobilization of EUGT11 and UGT76G1 on chitosan beads achieved 97.3% conversion of Reb A to Reb M with 72.5% activity retention after four cycles [195]. Similarly, co-immobilization of EUGT11 and sucrose synthase on Fe_3_O_4_@Uio-66 nanocomposites maintained 80% activity after eight cycles, demonstrating excellent reusability for Reb D production [196].

**Table 4:** Protein engineering and enzymatic synthesis of SGs and GSGs.

Sources	Enzyme	Protein Engineering	Engineering Effects	Key Strategies	Substrate	**Product**	**Yield**	**Reference**
stevia leaves	cellulase	-	-	cellulase/starch-mediated transglycosylation	Stv	Reb A	enriched Reb A content (66%)	[184]
*Irpex lacteus*	β-1,3-Glucanase	-	-	transglycosylation by *Il*β-1,3-Glucanase	Stv	Reb A	62.5% conversion to Reb A	[184,185]
*Stevia rebaudiana* *Acidithiobacillus caldus*	UGT76G1 *Ac*Susy	L637M-T640V	Enhanced affinity for UDP	Fusion of UGT76G1 and *Ac*Susy by linker	Stv	Reb A	18.8 ± 0.6 g/L	[197]
*Stevia rebaudiana*	UGT91D2	V155T	Enhanced activity in budding yeast and *Nicotiana benthamiana* towards Reb A	-	Reb A	Reb D		[186]
*Oryza sativa*	*Os*UGT91C1 (EUGT11)	F379A	2.18-fold increased catalytic efficiency towards Reb A	-	Reb A	Reb D		[188]
		F379A/F208M	Enhance the desirable β (1–2) glucosylation, eliminate β (1–6) glucosylation; 4-fold increased catalytic efficiency towards Reb A	Biochemical and structural characterization of *Os*UGT91C1	Reb A	Reb D	4-fold increased catalytic efficiency towards Reb A	[156]
*Solanum lycopersicum*	*Sl*UGTSL2	N358F	Enhance the desirable β (1–2) glucosylation (Reb D), eliminate β (1–6) glucosylation (side-product Reb M2); 1.6-fold of enhanced activity towards Reb A	Multi-enzyme reaction system with UGT76G1, *Sl*UGTSL2 and *St*SUS1	Stv	Reb D	14.4 g/L Reb D from 20 g/L Stv for 24 h	[157]
*Panax ginseng*	*Pg*UGT	A11L/F39Y/S55P/N109K/A250E/I279L/V304L/T329I	3.2-fold higher catalytic activity and enhanced thermostability (to 40 °C)	-	Reb A	Reb D		[158]
*Panax ginseng*	UGT94B1	I146G/P174V	Its catalytic efficiency toward Stv and Reb A is 4-fold and 3.1-fold that of the wild type, respectively	Cascade reaction with *At*SuSy and UGT76G1-M3	Stv, Reb A	Reb M	38.8 g/L with a 85.5% yield	[194]
*Bacillus subtilus* 168	*Bs*YojK	I241T/G327N	7.35-fold increase in catalytic activity	Cascade reaction with *At*SuSy	Reb A	Reb D	20.59 g/L with a 91.29% yield	[160]
		S158E/A218H/I241T/G327N/A369K	Enhanced thermos-stability and 1.39-fold increased activity at 50 °C	-	Reb A	Reb D	87.70% and up to 25 mM	[189]
		I241T/G327N/Q251M or I241T/G327N/R366P	The optimal temperature is 55 °C and remarkably enhanced thermo-stability at 50 °C	-	Reb A	Reb D	9.71 g/L	[190]
*Stevia rebaudiana*	UGT76G1	T146G or H155L	increased enzymatic activity and diminished side-product production in *Saccharomyces cerevisiae*	-	-	Reb D and Reb M	-	[33]
	UGT76G1	S195Q	1.2-fold and 2.0-fold increased catalytic efficiency toward Reb E and Reb D, respectively	Co-expressed *E. coli* cell lysate of UGT76G1 S195Q and *Mc*SuSy	Reb E	Reb D and Reb M	10.5 g/L Reb D 12.8 g/L Reb M	[198]
	UGT76G1	T284S	the crystal structures of SrUGT76G1 with multiple ligands; decrease the production of side product Reb I	Cascade reaction with *Os*UGT91C1 and *At*SuSy	Reb A	Reb M	-	[148]
	UGT76G1	T284S/M88L/L200A	reduce distances from Reb D to catalytic residues and UDPG; 2.38-fold increased activity compared with T284S mutant	-	Reb D	Reb M	23.37 mg/mL with a 90.50% yield	[191]
	UGT76G1	I30M/K53A/R141P/G349P/L200A/T284S/M88L	increased optimal temperature to 45 °C; 1.16-fold improvement of catalytic activity compared with T284S/M88L/L200A mutant	-	Reb D	Reb M	22.65 mM with a yield of 90.60% at 45 °C	[193]
	UGT76G1	L200A/L379M	10-fold increased enzymatic activity at 50 °C	-	Reb D	Reb M	45.05 g/L with a 96.85% yield at 50 °C	[192]
	UGT76G4	Q199I/ G200Y	1.46-fold increased enzymatic activity	-	Reb E	Reb M	13.62 ± 0.55 mM with a 55% yield	[151]
	UGT76G4	H155S/Q199I/G200Y	23-fold increased enzymatic activity	-	Reb D	Reb M	45.03 ± 1.92 mM with a 90% yield	[151]
*Oryza sativa* *Stevia rebaudiana*	*Os*EUGT11 UGT76G1	Co-immobilization on chitosan beads	higher activity (3.2-fold), stability	Purified protein from *E. coli*	Reb A	Reb M	72.2% yield, 4.82 g/L	[195]
*Oryza sativa* *Arabidopsis thaliana*	*Os*EUGT11 *AtSUS*	Co-immobilization on Fe_3_O_4_@Uio-66 nanocomposites	high reusability and improved storage stability	Purified protein from *E. coli*	Reb A	Reb D		[196]
*Bacillus subtilus* 168	*Bs*YjiC	-	-	Cascade reaction with *At*SuSy	Reb A	Reb L2	30.94 mg/mL	[167]
*Sesamum indicum*	*Si*UGT94D1	-	-	Cascade reaction with *At*SuSy	Reb A	Reb D2	10.69 mg/mL	[168]
*Oryza sativa*	*Os*EUGT11	-	-		Reb E	Reb D2′		[199]
*Sesamum indicum*	*Si*UGT94D1	F119I/D188P	6.33-fold increased activity towards Reb D	Cascade reaction with *At*SuSy	Reb D	Reb M2	29.79 mg/mL	[169]
*Nicotiana tabacum*	*Nt*UGT	F72L/L123P/L157P	5000-fold increased activity towards Reb D, enhanced thermostability	Cascade reaction with *Sl*UGTSL2 N358F and *At*SuSy	Reb A	Reb M2	78.8 g/L at 84.56% yield	[170]
*Gardenia jasminoides*	*Gj*UGT94E13	F169G/I185G	13.9-fold higher activity towards Reb D	Cascade reaction with *At*SuSy	Reb D	Reb M8	24.53 mM with 98% conversion	[166]
*Gardenia jasminoides*	*Gj*UGT94E13	F169A/I185A	12-fold higher activity towards Reb M2	Cascade reaction with *At*SuSy	Reb M2	Reb M9	42.8 g/L	[50]

#### 6.2.2. Microbial Synthesis

Microbial whole-cell biocatalysis represents a promising production paradigm, leveraging the cell’s innate capacity for cofactor regeneration and multi-enzyme cascade reactions to synthesize SGs from simple carbon sources. This platform offers the potential for a consolidated, fermentative “sugar-to-sweetener” process. Substantial foundational progress has been made in suitable microbial hosts such as *Escherichia coli* [146], *Saccharomyces cerevisiae* [138,159,200,201,202,203], and *Pichia pastoris* [163,204,205,206,207].

*Escherichia coli* has been engineered as a prokaryotic chassis for SG production, benefiting from its well-characterized genetics and rapid growth. As in Table 5, applying a modular engineering strategy, an SG biosynthetic pathway in *E. coli* was constructed by co-expressing key enzymes from the *ent*-kaurene and SG modules, along with a novel 13α-hydroxylase (KAH) and UDP-glucosyltransferase (UGT91D2w) identified via RNA-seq of *S. rebaudiana* [187]. Initial titers, however, remained low (10.03 mg/L Reb A) due to poor enzyme expression and host-cell incompatibilities [187]. Subsequent strategies focused on enhancing precursor flux and enzyme performance. Modular overexpression of terpenoid pathway genes (GGPPS, CPPS, KS) combined with precursor pathway enhancement (DXS, IDI, IspA) boosted *ent*-kaurene production to 578 mg/L [161]. Further optimization via 5′-UTR engineering of key genes and modulation of cofactor ratios elevated *ent*-KA titers to 50.7 mg/L [208]. For the downstream glycosylation steps, innovative approaches like Smt3-fusion and co-expression of chaperone-like proteins (PrpD, MalK) significantly improved the solubility of UGT76G1. This culminated in the highest reported *E. coli* titers to date: 4.8 g/L Reb A and 1.8 g/L Reb M in fed-batch fermentation [209], demonstrating the efficacy of integrated protein and metabolic engineering. This research demonstrated the potential of *E. coli* as a chassis for P450-dependent terpenoid pathways.

*Saccharomyces cerevisiae* is a preferred eukaryotic host for SG biosynthesis, combining the advantageous protein processing and folding capabilities of higher eukaryotes with the genetic and operational simplicity of prokaryotes. This balance facilitates cost-effective cultivation and high-level expression, enabling the establishment of de novo and reconstituted pathways for sustainable SG production from simple sugars [138,159,200,201,202,203]. A central strategy involves the modular design and optimization of the biosynthetic pathway, conceptually partitioning it into core functional units (terpenoid backbone, P450 oxidation, glycosylation, UDPG regeneration, and transport) for targeted engineering [200]. As in Table 5, this framework enabled systematic improvements: engineering P450 complexes (KO75/KAH82 with *Sr*CPR1) enhanced the conversion to steviol [138]; glycosyltransferases (UGT76G1, UGT91D2) were optimized and overexpressed [159,200]; and sucrose synthase overexpression facilitated internal UDPG recycling [201,202,203]. Complementary metabolic enhancements—such as knocking out hydrolytic enzymes (SCW2), overexpressing nucleotide sugar synthases (UGP1), regulating growth (via SIR2) [202], engineering efflux pumps (PDR11), and enhancing stress tolerance (MSN4) [200]—collectively boosted performance. These integrated efforts yielded impressive titers, including 1.92 g/L Rub [201], 5.27 g/L Reb D [159], and 12.5 g/L Reb M [202], underscoring the robust potential of engineered yeast for industrial SG synthesis.

*Pichia pastoris* is valued as a eukaryotic host for its strong, inducible promoters (e.g., AOX1) and high-density fermentation capability [204], making it suitable for whole-cell biocatalysis of SGs [163,205,206,207]. Co-expression of sucrose synthase (mbSUS) and UGT76G1 created an efficient whole-cell catalyst (Table 5), producing 261.2 mM Reb A within 26 h [207]. Optimized expression of *Os*EUGT11 enabled a 95.31% conversion of Reb A to Reb D [163]. A particularly notable advance involved the cell-surface display of a mutant *Pg*UGT (*Pg*M8) and mbSUS via GPI anchoring, creating a reusable whole-cell biocatalyst that produced 48.2 g/L Reb D in 33 h without cell disruption, highlighting its scalability [206]. Furthermore, a fusion enzyme (UGT76G1–linker–UGT91C1) improved substrate channeling and stability, enhancing Reb M (~0.24 mM) synthesis in *P. pastoris* [205]. These results position engineered *P. pastoris* as a versatile and efficient platform for high-yield SG production.

#### 6.2.3. Technological and Regulatory Comparison: Enzymatic vs. Microbial Production

The choice between enzymatic and microbial production of SGs involves a strategic trade-off. Enzymatic bioconversion utilizes engineered glycosyltransferases for high-yield, selective synthesis from plant-derived precursors, offering scalable near-term solutions, though it remains dependent on agricultural steviol supply. In contrast, microbial synthesis in engineered yeast aims for a consolidated “sugar-to-sweetener” process, promising greater sustainability and supply chain independence. While high titers have been reported (e.g., 48.2 g/L Reb D [206], 12.5 g/L Reb M [202]), this approach faces bottlenecks like UDP-sugar supply and host toxicity, which can increase complexity and cost [197]. These strategies are complementary: enzymatic conversion meets current market needs, while advances in synthetic biology are needed to unlock the full potential of microbial platforms [24].

Safety and regulatory frameworks for both production routes are well-established. Major markets classify SGs from these novel processes as new food additives, including China, EU, USA, Australia, and New Zealand, requiring pre-market safety assessments and specific quality standards. Regulatory clarity, such as the EU’s distinction between plant-derived (E 960a) [70,210] and enzymatically converted (E 960c) SGs [66,67], combined with transparent consumer education, is crucial for navigating “natural” labeling claims and ensuring market trust in next-generation SGs.

**Table 5:** Microbial biosynthesis of the precursors, SGs and GSGs.

Host	Key Strategies	Precursor	**Product**	**Titer/Yield**	**Reference**
*Escherichia coli* BL21	Co-overexpression of key enzymes of the *ent*-kaurene module, and DXS, IDI, and IspA for isoprenoid precursor enhancing		*Ent*-kaurene	578 mg/L	[161]
*Synechococcus elongatus*	Optimization of the CYP-CPR and KO-KAH-CPR combinations, utilizing photosynthetic bacteria to produce enantiomeric abietic acid from CO_2_.		*Ent*-KA	2.9 mg/L	[211]
*Escherichia coli* BL21	Modular expression of key enzymes of the *ent*-kaurene module, and overexpression of *Sr*KAHn2 and *Sr*UGT91D2w; N-terminal engineering of *At*CYP714A2; Assembly of the UGT module and combination with the CYP module.		*Ent*-KA	78.52 mg/L	[187]
			Steviol	15.47 mg/L	
			Reb A	10.03 mg/L	
*Escherichia coli* BL21	Over-expression of 5′UTR-engineered GGPPS, CPS, and KO; Enhancing the NADPH/NADP ratio and over-expression of N-terminal modified *Sr*KO; Over-expressing the fusion protein of UtrCYP714A2 and *At*CPR2.		*Ent*-kaurene	623.6 ± 3.0 mg/L	[208]
			*Ent*-KA	50.7 ± 9.8 mg/L	
			Steviol	38.4 ± 1.7 mg/L	
*Escherichia coli* BL21	Fusion expression with Smt3 and co-expression of endogenous prpD and malK to enhance the solubility of UGT76G1		Reb A	4.8 g/L	[209]
			Reb M	1.8 g/L	
*Saccharomyces cerevisiae*	Multiple species’ UDP-glycosyltransferases (*Rs*UGT85A57, *Ak*UGT75L21, *Sr*UGT85C2, and *Sr*UGT74G1) were coupled with sucrose synthase, and mutation of *Sr*UGT74G1 increased the yield of Rub.	Stv	S13G	0.45 ± 0.06 g/L	[201]
			Rub	1.92 ± 0.17 g/L	
*Saccharomyces cerevisiae*	Obtain the optimal combination of KO, KAH, and CPR enzymes, and express the recombinant pathway to produce steviol	Glucose	*Ent*-KA	<90 mg/L	[138]
			Steviol		
*Saccharomyces cerevisiae*	Establishment of a de novo biosynthetic pathway for Rub and SGs	Glucose	Rub	1368.6 mg/L	[200]
			Reb A	6.2 mg/L	
			Reb D	11.4 mg/L	
			Reb M	17.6 mg/L	
*Saccharomyces cerevisiae*	Whole cell bioreactor with constitutively over-expressed UGT76G1	Stv	Reb A	1.16 g/L	[203]
*Saccharomyces cerevisiae* YPH499	Cascade reaction involving *St*UGT and *Gs*SUS1, enhancing cell permeability	Reb A	Reb D	5.27 g/L	[159]
*Saccharomyces cerevisiae*	Knock-out endogenous glycosyl hydrolase SCW2, silencing information regulator 2 (SIR2) to prolong the growth cycle, overexpressing UGP1, and co-expressing UGT91D2 and UGT76G1	Stv	Reb M	12.5 g/L; a 77.9% yield	[202]
*Pichia pastoris*	Optimizing the gene dose ratio of mbSUS and UGT76G1 in a 1 L batch	Stv	Reb A	252.6 g/L (26h)	[207]
*Pichia pastoris*	Secretory expression of EUGT11		Reb D	95.31%	[163]
*Pichia pastoris*	Surface display of *Pg*UGTM8 with optimized copy number, and co-expressed with mbSUS	Reb A	Reb D	48.2 g/L	[206]
*Pichia pastoris*	Construction of a fusion enzyme of UGT76G1 and UGT91C1	Reb A	Reb M	~0.24 mM	[205]

## 7. Physicochemical and Sensory Improvement

### 7.1. The Structure–Property Relationship of SGs

The glycosylation pattern—specifically the number, position, and linkage of glucosyl units at the C-13 and C-19 positions of the steviol core—fundamentally dictates the sensory profile (sweetness, bitterness, and aftertaste) and solubility of SGs, and thereby governs key commercial limitations such as bitterness, licorice-like off-tastes, and poor solubility [30,38,212].

For designated SGs, increased glucosylation at the C-19 position attenuates both sweetness and bitterness perception. Nevertheless, C-13 glucosylationenhances sweetness while suppressing bitterness, a phenomenon attributable to differential steric accommodation within human sweet (hSTR) and bitter (hBTR) taste receptor binding pockets [185,213,214]. Structural analysis reveals that hSTR and hBTR possess distinctly configured binding sites: hSTR contains a wide, accessible binding cleft between domain lobes that accommodates heavily glycosylated SGs at the C-13 position, promoting strong binding via multipoint interactions and enhanced sweetness [213]. In contrast, hBTR features a narrow, sterically constrained site that only permits entry to minimally glycosylated moieties at C-19; additional glucosyl units at C-13 hinder binding, reducing perceived bitterness. Consequently, SGs with extended C-13 glycosylation (e.g., from Stv to Reb A, Reb D, and Reb M), within 3, exhibit high sweetness but low bitterness [214]. While extended glycosylation at C-19 (e.g., from RA to Reb M) does not impair hSTR binding, it disrupts hBTR interaction due to steric constraints, thereby diminishing both bitterness and overall sweetness perception [213]. The high-purity sweetness of Reb M arises from an optimal structural motif balancing extended glycosylation at both C-13 and C-19 positions, achieving maximal sweetness potency with minimal bitterness [30,185,215]. Beyond taste, glycosylation significantly affects the water solubility properties [216]. The addition of hydrophilic glucosyl moieties, particularly at the C-13/C-19 positions, can increase solubility [217,218], addressing a key limitation for certain applications.

Accordingly, enzymatic targeted glucosylation, thus, is a promising strategy to address SG limitations and generate glucosylated derivatives (GSGs) with refined sensory profiles and higher solubility, and has become a research hotspot.

### 7.2. Enzymatic and Regioselective Glucosylation for Property Improvement

Initial efforts primarily employed cyclodextrin glucanotransferases (CGTases) [219,220] or glucansucrase [217,221,222,223,224], which non-specifically add 1–6 glucosyl units to substrates like Stv [219,220,221] or Reb A [218,220,225,226]. While effective in reducing bitterness and astringency, the inherent lack of regioselectivity and chain-length control in CGTases or glucansucrase limits the precise and sufficient synthesis of well-defined GSGs [217,221,223,224,227]. Notwithstanding these limitations, the observed enhancement of water solubility and stability through α-glucosylation presents a notable advantage [217,218,223].

**Figure 5:**
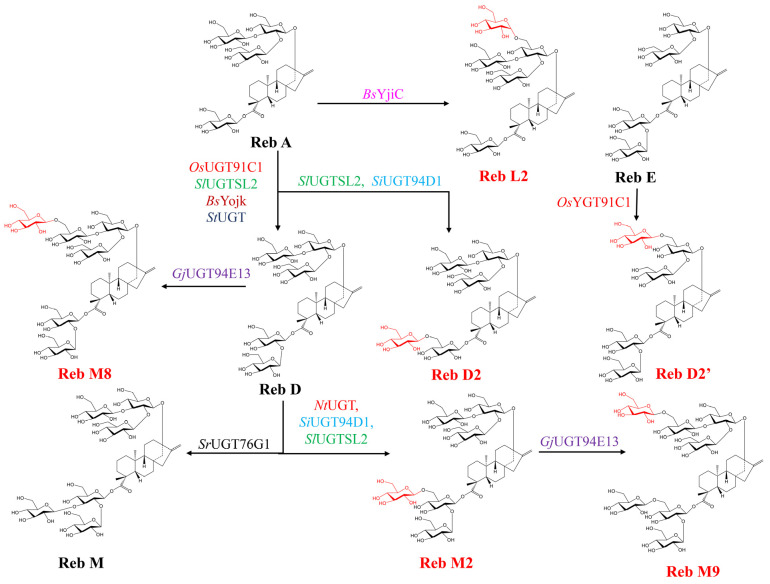
The structure and enzymatic synthesis of GSGs. *Si*, *Sesamum indicum*; *Nt*, *Nicotiana tabacum*; *Bs*, *Bacillus subtilis* 168; *Os*, *Oryza sativa*; *Gj*, *Gardenia jasminoides*; *Sr*, *Steruia rebaudiana*; *Sl*, *Solanum lycopersicum*. Red moieties: glycosylation sites. Red labels under the compounds: glycosylated steviol glycosides. Colored labels above arrows: enzymes (various sources) for each reaction.

Alternatively, UGTs catalyzing stereospecific glycosylation reactions have attracted more attention recently. A notable modification is β-1,6-glycosylation at the C-13 position, which is supposed to enhance sweetness, reduce bitterness, and improve hydrophilicity and solubility [217]—an advantage also observed in other sweet-tasting glycosides such as mogroside M5 [228]. This is exemplified by C-13-specific β-1,6-O-glycosylation: *Bs*YjiC converts Reb A to the sweeter Reb L2 (Figure 5), a derivative with heightened sweetness [167]; *Os*EUGT11 produces the isomer Reb D2′ from Reb E [199]; and *Gj*UGT94E13 yields the novel derivative Reb M8 from Reb D, noted for its superior sweetness and TNF-α inhibitory activity [43]. Concurrently, β-1,6-O-glycosylation at the C-19 position generates isomers with refined sensory profiles. For instance, Reb D2, a byproduct from Reb A conversion, can be selectively synthesized by *Si*UGT94D1 [168,229], and the serendipitously discovered isomer Reb M2 features a C-19 β-1,6-O-glycosyl linkage [49]. Reb M2 can be further glycosylated at C-13 by *Gj*UGT94E13 to produce Reb M9 (Figure 5) [50]. Both Reb M2 and Reb M9 exhibit dramatically purified, sucrose-like sweetness (200× and 300–450× sucrose, respectively), minimal bitterness, and enhanced solubility, showcasing substantial application potential (Table 2) [49,50,169]. While exploration of Reb D2 and its isomer Reb D2′ remains limited by uncharacterized taste and costly substrates, the precise production of other derivatives has been advanced through extensive enzyme engineering. A *Bs*YjiC/*At*SuSy cofactor regeneration system achieving 91.34% yield of Reb L2 [167]. Variants of *Si*UGT94D1(F119I/D188P) [169] and *Nt*UGT (F72L/L123P/L157P) [170] showed dramatic increases in catalytic efficiency for Reb M2 synthesis (Table 4). A one-pot system using *Sl*UGTSL2 N358F and *Nt*UGT F72L/L123P/L157P demonstrated scalability, converting 70 g/L Reb A into 78.8 g/L Reb M2 at 84.56% yield [170]. Similarly, semi-rational design of *Gj*UGT94E13 generated the hyperactive variant (F169A/I185A) that enables high-titer, gram-scale production of Reb M9 (42.8 g/L) from Reb M2 [50,166]. 

Thus, a key research direction is to discover and engineer glycosyltransferases for efficient, regioselective glucosylation at the C-13/C-19 positions to create tailor-made SGs with optimized properties. The underlying structure–property relationships also warrant further investigation [213,218].

### 7.3. Formulation and Assembly

To overcome the characteristic bitterness and limited solubility of SGs, formulation strategies involving blending with other sweeteners and advanced assembly techniques have been developed. Blending Reb A with flavor modulators such as neohesperidin dihydrochalcone (NHDC) can introduce pleasant botanical notes and enhance sweetness synergy, as evidenced in ternary systems with alitame, showing a 99.4% increase in sweetness intensity, though such pronounced synergism is less frequent in complex blends [230]. The effect of blending is strongly ingredient-dependent: maltitol suppresses the bitterness and astringency of Reb A, whereas erythritol, despite generally improving temporal sweetness and reducing aftertaste, may enhance bitterness at certain ratios [51,52,231,232]. Allulose complements SGs and mogrosides by neutralizing off-flavors, and its functional properties—such as low freezing point depression and Maillard reactivity—make it suitable for frozen and baked products. These blends exploit complementary physicochemical and receptor-level interactions (e.g., T1R2/T1R3 modulation) to enhance sweetness (synergy factor 1.5–2.5×) and mask undesirable notes, enabling clean-label, calorie-reduced products [233]. Industrial implementations include co-crystallization of SGs with erythritol for synchronized release and ternary blends (e.g., SGs-allulose-mogrosides) achieving >95% sensory similarity to sucrose. Time-intensity and descriptive analyses confirm that binary blends replacing 50–75% sucrose effectively mimic its temporal profile while minimizing off-flavors [234]. In vitro studies reveal that Reb A’s synergy with erythritol and thaumatin stems from selective TAS1R2/T1R3 activation with minimal TAS2R engagement, accounting for its low off-taste [235]. Assembly-based approaches, such as ternary Reb D-erythritol-fructose solid solutions, enhance solubility and taste [60]. Beyond passive blending, the intrinsic surfactant properties of certain SGs can be harnessed for active bitterness suppression. Stv, with its amphiphilic structure, can form mixed micelles with other bitter compounds, such as saponins, when its concentration exceeds the critical micelle concentration (CMC). This sandwich-like micellar structure encapsulates hydrophobic bitter moieties in its core, physically blocking their interaction with human bitter taste receptors (hT2R4/hT2R14), thereby reducing bitterness perception [236,237,238,239]. This self-assembly behavior, also observed in structures like micelles and rods formed by Reb A in aqueous systems, not only improves sensory properties [240,241], but also offers potential for functional encapsulation [238].

## 8. Commercialization of SGs

Japan pioneered the commercial use of SGs as early as 1971. The global market for natural sweeteners has seen exponential growth, reaching JPY 22.49 billion in 2022 and projected to reach JPY 24.7 billion by 2025. SGs constitute nearly 5% of the natural sweeteners market, yet account for less than 20% of sugar replacements. With rising obesity and diabetes rates, there is an inevitable trend towards reduced-sugar diets. The market outlook for SGs is optimistic, with anticipated demand reaching only 100,000 tons by 2035. Industry forecasts suggest the future alternative sweeteners market could grow to USD 100 billion. In recent years, major international food and beverage companies like PepsiCo have launched products such as PureVia, based on SGs, featuring beverages containing Reb A [242]. SGs, as high-intensity, low-calorie sugar substitutes, are increasingly favored by food and beverage manufacturers. Approval for SGs as food additives in key markets such as the US, EU, and Japan has established a legal and regulatory foundation for commercial applications. Technological advancements enhancing the purity and stability of SGs continue to drive new product development and market expansion, promising significant future prospects.

## 9. Future Perspectives

SGs represent powerful sweeteners to replace sugar, combining sucrose-like sensory properties with multifunctional health benefits. To realize the low-cost and scalable production of diverse SGs, concerted efforts are required in the following areas.

### 9.1. Advancing Next-Generation Enzymes and Pathway Engineering

As discussed in Section 6.2.3 for robust enzymic and microbial synthesis of SGs, key huddles include the following: the catalytic efficiency and specificity, the solubility and the stability of core glycosyltransferases (e.g., UGT76G1/4) involved in SG biosynthesis, the insufficient intracellular supply of the essential sugar donor UDPG, the inherent host toxicity imposed by hydrophobic pathway intermediates, and the balancing of the host metabolic flux.

Future progress may depend on a synergistic, multidisciplinary strategy integrating synthetic biology, AI-guided design, and structural biology. This integrated approach is essential for optimizing the entire production system, from the molecular performance of individual enzymes to the coordinated function of complex metabolic networks. At the molecular level, structural biology provides the foundational insights for the rational engineering of key enzymes, such as glycosyltransferases, to enhance their catalytic efficiency, stability, and specificity. AI dramatically accelerates this process by mining genomic data to discover novel enzyme variants [243], by predicting the protein monomer structures and accurately modeling protein–small molecule complexes (e.g., AlphaFold 3) [244], and the function-enhancing mutations [245]. Beyond single enzymes, AI-driven tools are transformative for systems-level optimization. Techniques like deep learning and neural networks can model complex biological dynamics to optimize genetic circuit parameters [243,245], discover efficient metabolic network topologies, and assess overall system robustness.

Embedding these predictive models into iterative Design-Build-Test-Learn (DBTL) cycles will significantly accelerate the development of high-performance microbial cell factories. Ultimately, the refactoring of de novo biosynthetic pathways in robust microbial chassis—guided by these AI-enhanced designs and coupled with dynamic metabolic regulation—will be key to alleviating metabolic burden, boosting the titers of high-value minor glycosides (e.g., Reb M, Reb D), and achieving commercially viable production costs.

### 9.2. Enhancing Green and Scalable Bioprocessing

Achieving sustainable and economically competitive production requires parallel innovations in bioprocessing. Scaling up SG manufacturing presents a key challenge in minimizing environmental impact and energy consumption. Advancing greener extraction techniques, such as Natural Deep Eutectic Solvents (NADES) and supercritical CO_2_ extraction, alongside the development of efficient, integrated biocatalytic systems, will be essential. The implementation of immobilized enzymes and multi-enzyme cascade reactions, particularly those synergistically coupled with sucrose synthase for in situ UDP-glucose recycling, exemplifies a path toward more efficient and reusable processes. Beyond process intensification, a fundamental shift in feedstock strategy is emerging. The exploration of alternative, non-sugar carbon sources, such as methanol and CO_2_ [211], as one-carbon feedstocks for engineered microbes, offers a promising route to circumvent the constraints and price volatility associated with conventional agricultural raw materials. This diversification of feedstocks paves the way for a more resilient, cost-effective, and truly sustainable foundation for the biomanufacturing of SGs.

### 9.3. Driving Function-Driven Product Development and Market Adoption

Beyond production, future success hinges on optimizing product profiles and expanding applications. SG formulations should leverage synergistic blending (e.g., with erythritol or allulose) to mask lingering off-flavors like bitterness and create well-rounded taste experiences, a key factor for consumer acceptance. Concurrently, clinical validation of their dose-responsive bioactivities—including antihypertensive and antidiabetic effects—will support their use in targeted functional foods and health products. Navigating the evolving global regulatory landscape for novel production methods (e.g., enzyme conversion, fermentation) and ensuring clear “natural” labeling will also be crucial for market access and building consumer trust.

### 9.4. Summary

In summary, SGs are promising sugar alternatives, combining growing sensory acceptability, regulatory safety, and potential health benefits. As consumer demand continues to shift toward natural and health-oriented products, SGs are well-positioned to drive future innovation in the food industry. However, realizing their full potential requires overcoming critical production challenges—particularly in scaling the biosynthesis of high-value minor glycosides (e.g., Reb M). The field is being transformed by advances in synthetic biology and AI, which are shifting the paradigm from simple extraction to precision biomanufacturing. Overcoming the remaining challenges in scaling the biosynthesis of high-value minor glycosides through interdisciplinary research will be key. By bridging the gap between laboratory innovation and industrial implementation, SGs can evolve beyond sweetening agents into versatile, high-value ingredients for a healthier and more sustainable food system.

## Figures and Tables

**Table 1:** Molecular formula and R1/R2 group composition of SG compounds.

Compound	Molecular Formula	R1	R2
Steviol	C_20_H_30_O_3_	H	H
Stv	C_38_H_60_O_18_	β-Glc-β-Glc(2→1)	β-Glc
SB	C_32_H_50_O_13_	β-Glc-β-Glc(2→1)	H
Rub	C_32_H_50_O_13_	β-Glc	β-Glc
Reb A	C_44_H_70_O_23_	β-Glc-β-Glc(2→1) 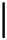 β-Glc(3→1)	β-Glc
Reb B	C_38_H_60_O_18_	β-Glc-β-Glc(2→1) 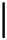 β-Glc(3→1)	H
Reb C	C_44_H_70_O_22_	β-Glc-α-Rha(2→1) 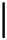 β-Glc(3→1)	β-Glc
Reb D	C_50_H_80_O_28_	β-Glc-β-Glc(2→1) 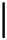 β-Glc(3→1)	β-Glc-β-Glc(2→1)
Reb E	C_44_H_70_O_23_	β-Glc-β-Glc(2→1)	β-Glc-β-Glc(2→1)
Reb F	C_43_H_68_O_22_	β-Glc-β-Xyl(2→1) 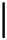 β-Glc(3→1)	β-Glc
Reb G	C_38_H_60_O_18_	β-Glc 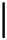 β-Glc(3→1)	H
Reb Q	C_44_H_70_O_23_	β-Glc 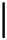 β-Glc(3→1)	β-Glc 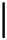 β-Glc(3→1)
Reb I	C_50_H_80_O_28_	β-Glc-β-Glc(2→1) 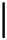 β-Glc(3→1)	β-Glc 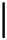 β-Glc(3→1)
Reb M	C_56_H_90_O_33_	β-Glc-β-Glc(2→1) 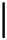 β-Glc(3→1)	β-Glc-β-Glc(2→1) 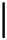 β-Glc(3→1)
Dulcoside A	C_38_H_60_O_17_	β-Glc-α-Rha(2→1)	β-Glc

**Table 2:** The main properties of some focused on SGs.

SGs	Sweetness (Times of Sucrose)	Flavor Profile	Solubility	Content in *S. rebaudiana*	**Bioactivity**	**Reference**
Rub	100–200	A clean, comfortable sweetness, minimal bitter/astringent aftertaste at low use levels, intensified bitterness at high levels	Poor water solubility	<5%	Neuroprotection and anti-inflammation; modulating gut microbiota and blood glucose	[25]
Stv	300	Bitterness with an unpleasant metallic aftertaste	Soluble in water, ethanol, and methanol; insoluble in organic solvents such as benzene, ether, and chloroform	5–10%	Anti-inflammatory, glycolipid metabolism regulation, and anti-cancer	[37]
Reb A	450	Rapid-onset sweetness, initial bitterness, lingering bitter-sweet aftertaste, clean, no off-flavor, non-grassy	Soluble in methanol, low solubility in water	2–5%	Anti-inflammation, glycolipid metabolism regulation, and anti-cancer	[9,38]
Reb B	150	Slow-onset, deficient sweetness with a lingering bitter-sweet aftertaste, clean, no off-flavor, non-grassy	Relatively poor water solubility	0.4–0.5%	Inhibiting cellular apoptosis and rebalancing the inflammatory response	[39]
Reb C	40–60	prolonged after-bitterness	N/A	<1%	N/A	[40]
Reb D	200–350	Rapid-onset, sucrose-like sweetness, long-lasting sweet aftertaste. Clean profile: non-bitter, no off-flavor, non-grassy	Poor water solubility	<0.2%	Anti-inflammation, increased glycolipid metabolism	[38,41,42,43]
Reb E	150–300	N/A	Slightly soluble in water	<1%	N/A	[44]
Reb F	25	N/A	N/A	<1%	N/A	[45]
Reb M	200–350	Fast-onset, sucrose-like sweetness, long sweet aftertaste, bitter-free, no off-flavor or grassiness	Slightly soluble in water.	<0.1%	improved insulin sensitivity, decreased weight gain	[3,38,42,46]
Reb I	170	N/A	N/A	N/A	N/A	[47]
Dulcoside A	50	A significant bitter aftertaste due to a rhamnose group	N/A	N/A	N/A	[48]
Reb M2	200	Excellent initial burst, sucrose-like sweetness, short aftertaste, soft mouthfeel, and clean finish, no bitterness or licorice off-taste	Better solubility in water	N/A	N/A	[49]
Reb M8	Comparable with Reb D	N/A	Better solubility in water	N/A	inhibitory effects on inflammatory factor TNF-α	[43]
Reb M9	300–450	Pure sweetness, barely perceptible bitterness	Better solubility in water than Reb M2	N/A	N/A	[50]

Note. N/A indicates not reported.

## Data Availability

No new data were created or analyzed in this study. Data sharing is not applicable to this article.

## Abbreviations

The following abbreviations are used in this manuscript:

*Ent*-KA	*Ent*-kaurenoic acid
SB	Steviolbioside
S-1,3-B	Steviol-1,3-bioside
S13G	Steviolmonoside
S19G	Steviol-19-O-β-D-glucoside
Rub	Rubusoside
Stv	Stevioside
Reb A	Rebaudioside A
Reb B	Rebaudioside B
Reb C	Rebaudioside C
Reb D	Rebaudioside D
Reb E	Rebaudioside E
Reb F	Rebaudioside F
Reb G	Rebaudioside G
Reb Q	Rebaudioside Q
Reb M	Rebaudioside M
Reb I	Rebaudioside I
Reb AM	Rebaudioside AM
GGPS	Geranylgeranyl Diphosphate Synthase
CPPS	Copalyl Diphosphate Synthase
KS	*Ent*-kaurene Synthase
KO	Kaurene Oxidase
Glc	Glucose
Xyl	Xylose
Rha	Rhamnose
